# Striatal ensembles continuously represent animals kinematics and limb movement dynamics during execution of a locomotor habit

**DOI:** 10.1186/1471-2202-14-S1-P325

**Published:** 2013-07-08

**Authors:** Pavel E Rueda-Orozco, David Robbe

**Affiliations:** 1 INSERM, U901; Aix-Marseille University, UMR 901; INMED, France

## 

The sensorimotor striatum contributes to the normal execution of motor habits but the mechanisms underlying this function are largely unknown[1]. Motor habits are stereotyped sequences of movements learned through a long trial-and-error process, automatically triggered by a set of sensory cues and that tend to persist despite outcome degradation (e.g. reward omission). We found that rats running on a treadmill become proficient in a fixed time interval estimation task by developing a highly stereotyped locomotor routine. Consistently with the definition of habits, the routine was acquired slowly (at least 2 months of daily practice), and once learned, it persisted for several sessions when the rewarding outcome was omitted. We took advantage of this unexpected behavior and used tetrode arrays to record the spiking activity of dorsolateral striatal ensembles while rats perform the locomotor habit. We report sequential activations of striatal neurons during the entire execution of the task. Importantly, we found that the firing rate of a large fraction of neurons was either locked to the locomotor limb movements or correlated with the kinematics of the habit (running speed, acceleration, position and time). These results contrast with the long-standing view that striatum is mainly concerned with action selection[2]. Rather movements and task kinematics encoding suggest that the striatum continuously control the execution of habitual action. Additional experiments are currently being performed to further investigate this hypothesis.

## References

[B1] Redgrave P Rodriguez M Smith Y Rodriguez-Oroz MC Lehericy S Bergman H Agid Y DeLong MR Obeso JA Goal-directed and habitual control in the basal ganglia: implications for Parkinson's disease Nat Rev Neurosci 2010 11 760 772 10.1038/nrn2915 20944662 PMC3124757

[B2] Calabresi P Di Filippo M Neuroscience: Brain's traffic lights Nature 2010 466 449 10.1038/466449a 20651682

